# Comparison of Behavioral Time Budget and Welfare Indicators in Two Local Laying Hen Genotypes (Atak-S and Atabey) in a Free-Range System

**DOI:** 10.3390/ani12010046

**Published:** 2021-12-27

**Authors:** Arda Sözcü, Aydın İpek, Züleyha Oğuz, Stefan Gunnarsson, Anja B. Riber

**Affiliations:** 1 Department of Animal Science, Faculty of Agriculture, Bursa Uludağ University, Bursa 16059, Turkey; aipek@uludag.edu.tr; 2 Poultry Research Institute, Republic of Turkey Ministry of Agriculture and Forestry, Ankara 06560, Turkey; zuleyha.oguz@tarimorman.gov.tr; 3 Department of Animal Environment and Health, Swedish University of Agricultural Sciences (SLU), 53223 Skara, Sweden; stefan.gunnarsson@slu.se; 4 Department of Animal Science, Aarhus University, 8830 Aarhus, Denmark; anja.riber@anis.au.dk

**Keywords:** behavior, dermatitis, free range, genotype, keel bone damage, welfare

## Abstract

**Simple Summary:**

It is known that laying hens kept in cage systems without access to enrichment have more welfare and behavioral problems. Therefore, alternative systems for egg production have gained popularity, e.g., free-range and organic systems, as they improve the birds’ possibilities to perform important specific behavior and thereby increase the welfare conditions in commercial farms. This study aimed to compare of the behaviors and welfare of two layer genotypes used in Turkey, Atak-S (brown) and Atabey (white), in a free-range system from 19 to 72 weeks of age. We evaluated multiple welfare indicators, including behavioral time budget, fear level, plumage condition, keel bone damage, and other body lesions. The birds were scored at 24, 40, 56, and 72 weeks of age. The Atabey hens showed more preening, walking–standing, and resting behavior, and they had a longer duration of tonic immobility. The Atak-S hens tended to perform more feather pecking and explorative pecking, and they had more foot lesions, plumage damage, skin injuries, and keel bone damages. Current results can be beneficial for the choice of genotype to use in free-range systems.

**Abstract:**

Free-range systems are considered to improve bird health and welfare, thereby satisfying consumer demands. Behavioral time budget, fear level and clinical welfare indicators were compared for two Turkish laying hen genotypes, Atak-S (brown) and Atabey (white), reared in a free-range system. A total of 420 laying hens (210 Atak-S, 210 Atabey) were studied between 19 and 72 weeks of age. Higher percentages of eating and drinking behavior, feather pecking, and explorative pecking were observed for Atak-S hens, whereas Atabey hens were preening, walking–standing, and resting more. The duration of tonic immobility was longer, and the number of inductions was lower in Atabey compared with Atak-S hens. Atabey hens had less keel bone damages and better plumage conditions on the breast, wing, and tail at 56 and 72 weeks of age than Atak-S hens. Footpad dermatitis was more common in Atabey hens at 40 weeks, whereas Atak-S hens had a higher prevalence of footpad dermatitis with moderate lesions at 72 weeks of age. These findings indicate that free-range Atak-S hens may be more prone to keel bone damage and development of feather pecking, but they showed less foot lesions and were less fearful.

## 1. Introduction

Continuous selection of laying hens has resulted in higher egg production per laying period. However, this has been linked to an increase in behavioral and welfare-related problems in laying hens [1]. Additionally, the space limitation and lack of resources in unfurnished cage systems, causing physical and behavioral restriction, may result in serious health and welfare problems during the production period [2]. These issues have caused European consumers to object to cage egg production. As a result, unfurnished cages in laying hen production were banned by the European Union (EU) from January 2012 [3]. Since then, the demand has increased for non-cage systems, e.g., free-range and organic systems. These systems aim to increase animal welfare and satisfy consumer demands for protection of the laying hens and the environment [4]. Furthermore, the European Commission has announced the preparation of a legislative proposal by 2023 to prohibit housing of farm animals in cages in the EU from 2027.

Behavioral observations of hens can be used to assess their welfare status [5]. Recent studies have examined the interaction between welfare traits and behavioral patterns, as well as the productivity of laying hens in sustainable production systems [6,7]. When hens have access to outdoor areas, more natural behavior, including foraging activity, may be exhibited [4]. However, access to outdoor areas may also impair bird welfare status, due to higher risks of parasite infestation and predation. Declines in productivity in outdoor systems, caused by increased mortality, decreased feed efficiency, and reduced egg production, have been reported [4,8]. Previous research in Germany has demonstrated higher mortality rates in production systems with outdoor access than in unfurnished cages [9]. Lambton et al. [10] reported a higher prevalence of severe feather pecking (up to 85.6%) in organic and free-range production systems in the UK, when assessed using either direct behavioral observations or scoring of plumage condition.

To optimize hen welfare and productivity in free-range systems, the choice of genotype used is of crucial importance [6]. Local genotypes may have an advantage in this regard, by being adapted to local weather conditions. Studies comparing the welfare, including behavior, health, and productivity, of different genotypes can be useful for producers when choosing the most suitable genotype to achieve successful and profitable production. The production parameters, egg quality parameters, and yolk fatty acid profiles of Atak-S and Atabey genotypes have been reported by Sozcu et al. [11] as the first part of the FreeBirds project. These genotypes have been gaining importance in free-range and organic systems due to their potential to produce healthier eggs, protect the health and welfare status of laying hens, and contribute to an improvement of environmental protection aspects and sustainability. These genotypes are accepted as the first local hybrids of laying hens in Turkey and both Atak-S and Atabey are adapted to the local climatical conditions. According to the standard performance data for Atak-S and Atabey, under optimum management standards in cage systems, hen-day and hen-house egg production levels are 83.3 and 82.4%, respectively, for Atak-S, and 83.9 and 82.8%, respectively, for Atabey [11]. This study aimed to compare the behaviors and welfare of two Turkish genotypes of laying hens, Atak-S (brown) and Atabey (white), in a free-range system. Multiple welfare indicators, including behavioral time budget, fear level, plumage condition, keel bone damage, and presence of body abnormality or lesions, were assessed between 19 and 72 weeks of age.

## 2. Materials and Methods

### 2.1. Animals and Housing

A total of 420 laying hens of two local layer genotypes, 210 Atak-S (brown) and 210 Atabey (white), both developed by the Poultry Research Institute in Turkey, were used in the experiment. The birds were placed in the system at 19 weeks of age and the experiment ended at 72 weeks of age. The experimental design consisted of two genotypes with three subgroups (*n* = 3 pens/genotype, 70 hens/pen) used as replicates of each genotype. Upon arrival, all hens were individually weighed on a digital scale with precision ±0.1 g to determine body weight, and then randomly allocated to the pens, each measuring 3 m × 7 m.

The hens were kept in a free-range system designed to comply with the minimum standards of EU Directive 1999/74/EC [3]. Circular plastic feeders (6.3 cm feeder space per hen) and plastic bell drinkers (4.5 cm drinker space per hen) were provided indoors, where the floor was covered with wood shavings as litter. Perches (18 cm of perch length per hen) and nesting boxes (3.5 hens per nesting box) were available indoors and the indoor stocking density was 0.30 m^2^/hen. The free-range area (350 m^2^ for each pen) was covered by pasture and enclosed by wire fences to keep out predators and had a shelter. The outdoor stocking density was provided as 5 m^2^/hen. At the start of the experiment, the vegetation consisted of 60% perennial ryegrass (*Lolium perenne*), 10% white clover (*Trifolium repens*), and 30% alfalfa (*Medicago sativa*).

The indoor lighting regime was gradually increased by 1 h per week, from 14 h per 24 h period at 19 weeks of age to 16 h per 24 h period from 20 weeks to the end of the experimental period. The mean of temperature and relative humidity was 15.8 °C and 50.6%, respectively during the experimental period [11]. A standard layer diet for free-range systems (17.86% CP and 2750 ME kcal/kg) was used between 19–40 weeks of age and a different standard diet (16.45% CP and 2800 ME kcal/kg) was used between 41–72 weeks of age that was detailed in a previous study by Sozcu et al. [11].

### 2.2. Data Collection

In determining the behavioral time budget of the hens, the back of three randomly sampled focal birds from each pen was marked with green paint. Marking of focal birds was repeated frequently during the experiment. Behavioral observations were performed on four occasions during the experimental period (at 24, 40, 56, and 72 weeks of age), each time for 2 × 3 days, giving a total of 24 observation days. The live observations were made during the periods 9.00–10.00 h and 15.00–16.00 h, by the same observer, in indoor and outdoor areas. The observer sat or stood in a position outside the pens with a clear view of the entire pen under observation. Each pen was scanned at 5 min intervals, thus giving 12 records per pen. The numbers of birds in a pen performing each of the behaviors eating, drinking, preening, feather pecking, walking–standing, explorative pecking, and resting were sequentially recorded as a series of instantaneous scans. Corresponding scans were then made for the other five pens in turn. The definitions of behaviors used were modified from Zhao et al. [12] (Table 1).

A total of 36 hens per genotype (12 from each pen) were randomly selected and tested individually for tonic immobility (TI) response at 24, 40, 56, and 72 weeks of age. To measure the duration of TI, hens were caught randomly and carried to a separate room. A few seconds after the hen was caught, TI was induced according to Ghareeb et al. [13]. The maximum duration was set to 600 s.

A range of clinical welfare indicators was assessed for each hen at 24, 40, 56, and 72 weeks of age using the Welfare Quality protocol [14]. Comb condition was evaluated for abnormalities, including black or blue areas, dried areas, or pale comb. Comb pecking wounds were scored on a 3-point scale, where 0 was no wounds, 1 was one or two wounds, and 2 was three or more wounds on the comb. Plumage damage was determined by scoring three parts of the hen’s body (breast, wing, and tail) separately, using a 3-point scoring system where 0 was no or slight wear, 1 was moderate wear (<5 cm), and 2 was at least one featherless area > 5 cm in diameter. The scores for each body part were combined to give a total plumage score. Enlarged crop, respiratory infection, eye pathologies, enteritis, and toe damage were scored as 0 (absent) or 1 (present). Skin lesions were scored as 0 (<3 pecks or scratches), 1 (lesions < 2 cm or >3 pecks or scratches), or 2 (at least one lesion > 2 cm). Footpad dermatitis and hock burns were scored using a 3-point scale scoring system where 0 was feet intact and no abnormality, 1 was small lesions, necrosis, or proliferation of epithelium, but no or moderate swelling, and 2 was visible inflammation and swelling of foot or hock, respectively. Keel bone damage was assessed with palpation to detect keel bone damages, scored 0 for no abnormalities or 1 for deviation and 2 for fracture present.

To determine the relationship between keel bone damage and egg-breaking strength, a total of 30 eggs from each genotype were randomly sampled at 24, 40, 56, and 72 weeks of age. Eggshell breaking strength (kg/cm^2^) was measured for these eggs using an eggshell force reader (Orka Food Technology, Herzliya, Israel).

### 2.3. Statistical Analysis

All data were analyzed with the mixed model procedure in the statistical analysis software SAS (version 9.4, 2012, Cary, NC, USA). For the behavioral data and clinical welfare indicators analyses, the main effects (G—effect of genotypes and A—effect of age) and the combined effect (G × A interaction) were determined. Analyses of percentage data were conducted after arcsine square root transformation of the data. The time budget was calculated per pen as number of observations of each behavior as a percentage of the total number of observations for each hen age. Non-parametric data were analyzed with the Kruskal–Wallis test. Significant differences between means were compared using the Tukey test and were considered statistically different at *p* < 0.05. The effects of keel bone damage level on eggshell breaking strength and prevalence of feather pecking were determined by contrast analysis using the GLM procedure. Orthogonal polynomial contrast was applied to determine the linear responses to different levels of keel bone damage.

## 3. Results

The percentages of the different behaviors performed by the two genotypes (Atak-S and Atabey) are shown in Table 2. There was considerable variation in the behavioral time budget between genotypes and between hen ages. Higher percentages of eating (18.4 vs. 17.1%), drinking (5.2 vs. 3.7%), feather pecking (5.6 vs. 5.1%), and explorative pecking (8.0 vs. 5.3%) were observed for Atak-S compared with Atabey hens. A higher percentage of preening (3.8 vs. 2.8%), walking–standing (22.2 vs. 19.1%), and resting (42.9 vs. 41.0%) was observed for Atabey compared with Atak-S hens (*p* < 0.001). Observations of eating, drinking, and explorative pecking behaviors showed a significant increase with hen age (*p* < 0.001). In addition, the percentages of hens performing feather pecking and walking–standing were found to be lowest at 24 weeks of age. The percentage of resting showed a decline with age, from 47.3% at 24 weeks to 38.2% at 72 weeks (*p* < 0.001).

The results from the TI tests by the two genotypes (Atak-S and Atabey) are shown in Table 3. No significant interactions (genotype × age) were observed for number of inductions necessary to induce tonic immobility, or duration of tonic immobility. The duration of tonic immobility was longer (120.4 vs. 103.2 s; *p* < 0.001) and the number of inductions was lower (1.1 vs. 1.2; *p* < 0.001) in Atabey hens than in Atak-S hens.

**Figure 1:**
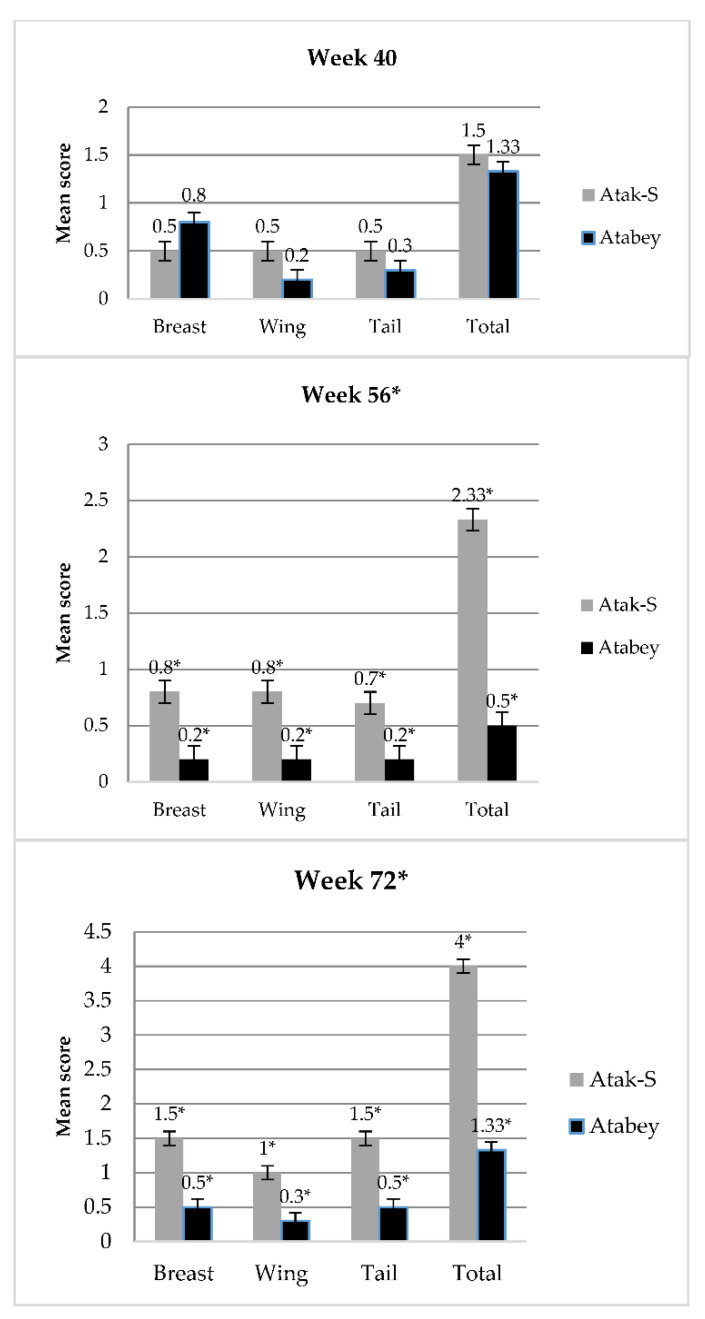
Plumage condition (mean score) of individual body part, by age (week 40, 56, and 72) and genotype (Atak-S or Atabey). Bars represent mean ± SE. (* *p* < 0.01, *n* = 70 laying hens/pen).

An effect of genotype on clinical welfare indicators was observed (Table 4). Atak-S hens displayed more comb pecking wounds at 24, 40, 56, and 72 weeks of age compared with Atabey hens (*p* < 0.001). At 24 weeks of age, no plumage damage on the breast, wing, and tail was observed for hens from either genotype. At week 40, no difference was found in plumage condition between the two genotypes (Figure 1). Meanwhile, Atabey hens had better plumage conditions on the breast, wing, and tail at 56 and 72 weeks of age compared with Atak-S hens (*p* < 0.001; Figure 1). Atak-S hens had more skin lesions at 40, 56, and 72 weeks of age than Atabey hens (0.37 vs. 0.17%; 0.67 vs. 0.33%, and 1.17 vs. 0.67%, respectively; *p* < 0.05). Footpad dermatitis prevalence was higher (0.33 vs. 0.17%; *p* < 0.001) in Atabey at 40 weeks of age, whereas Atak-S hens had a higher prevalence of footpad dermatitis at 72 weeks of age than Atabey hens (1.50 vs. 0.67%; *p* < 0.001). Apart from at 40 weeks of age, Atak-S hens had a higher prevalence of hock burns during the experimental period than Atabey hens (*p* < 0.01). The severity of hock burns increased with age, from 0.33 to 1.67% in Atak-S and from 0.17 to 1.17% in Atabey. During the experimental period, toe damage was more frequently observed in Atak-S than Atabey hens (*p* < 0.001). Likewise, keel bone damage was found to be more prevalent in Atak-S than Atabey hens at 56 and 72 weeks of age (*p* < 0.001).

**Figure 2:**
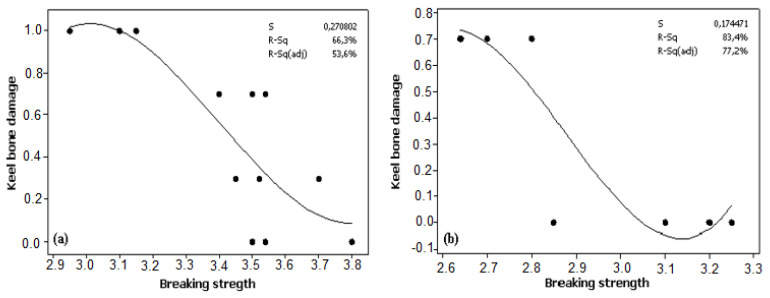
Linear regression of the relationship between keel bone damage and egg breaking strength in (**a**) Atak-S hens (*p* = 0.002) and (**b**) Atabey hens (*p* < 0.001).

**Figure 3:**
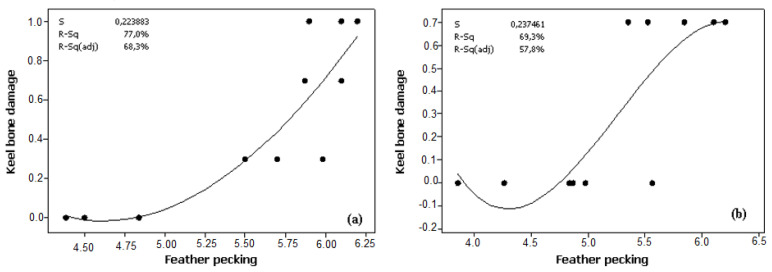
Linear regression of the relationship between keel bone damage and the prevalence of feather pecking in (**a**) Atak-S hens (*p* = 0.001) and (**b**) Atabey hens (*p* = 0.002).

The results showed a significant association between keel bone damage and eggshell breaking strength in Atak-S (*p* = 0.002) and Atabey (*p* < 0.001) (Figure 2). While keel bone damage increased with hen age, eggshell breaking strength declined linearly for both Atak-S and Atabey eggs. In both genotypes, the prevalence of feather pecking showed a positive relationship with keel bone damage (*p* < 0.001) (Figure 3).

## 4. Discussion

The results obtained in this study indicated that the genotype and age of laying hens in the free-range system affected their behavioral time budget. The most frequently recorded activities were resting, walking–standing, and eating in both genotypes and at all ages. Drinking is related to feed consumption and thus eating behavior [15]. In this study, drinking activity was higher in Atak-S hens, which showed a higher percentage of eating activity. Furthermore, both eating and drinking behavior increased with age, in parallel with increasing nutritional requirements arising from increasing body weight and egg production.

Preening helps laying hens keep their plumage in good condition. During preening, birds disperse preen oil, a lipid secretion from the preen gland [16], to the whole plumage. Our results showed a higher percentage of preening in Atabey hens and less plumage damage than in Atak-S hens. This may indicate that Atabey hens were more comfortable and had better welfare status in the free-range system compared to Atak-S hens. Previous studies by Lambton et al. [10] and Chielo et al. [17] found a positive relationship between range usage and better plumage condition.

Fear is an emotional state that can cause behavioral and physiological changes [18]. Such changes could act as reliable measures of fear level of hens, and thus as an indicator of their welfare status [4]. Fearfulness in hens is often assessed by measurement of tonic immobility, with a longer duration of tonic immobility indicating higher levels of fearfulness [19]. We found that Atabey hens reacted more fearfully, based on the tonic immobility test.

Our results demonstrated that Atak-S hens had a higher prevalence and more severe comb pecking wounds, plumage damages, and skin lesions. The former could be related to aggressive behavior, and the feather pecking observed in Atak-S hens may have caused skin lesion in addition to plumage damage, either directly during the pulling of feathers or indirectly due to a development into injurious pecking at the skin. A link between feather pecking and cannibalistic pecking causing skin injuries has been found in previous studies (e.g., Cloutier et al. [20], Lambton et al. [21].

Footpad pad disorders are frequently reported in non-cage egg production systems e.g., [22]. Atak-S hens had a higher prevalence of footpad dermatitis, hock burns, and toe damage. However, the severity of these lesions was low, approximately a score of 1.50 at 72 weeks of age. This mainly reflected hyperkeratosis or minor lesions, necrosis/proliferation of epithelium, or slight swelling/inflammation of the footpad or hock. The results showed that Atak-S hens were more susceptible to foot health issues in the free-range system than Atabey hens. This is in agreement with previous findings by Tauson and Abrahamsson [23], and Heerkens et al. [22] for other commercial brown and white genotypes (e.g., DeKalb XL and LSL, Shaver 288 and 744, ISA Brown, and LSL layers). Furthermore, our results clearly indicated that the prevalence and the severity of these lesions increased with increasing hen age.

Keel bone deformation is a well-known welfare problem in laying hens [24,25]. Strong evidence exists that keel bone deformation involving fractures causes pain in the laying hens [26]. In the present study, Atak-S hens had more severe keel bone damage at all ages studied. The severity of the damage observed suggests that these Atak-S hens had impaired welfare. Our results also showed that the incidence and severity of keel bone damage increased with age in both genotypes. Similar findings have been found for laying hens in different production systems [27,28,29,30].

One causal explanation for keel bone damage is poor bone quality related to osteoporosis, due to inadequate dietary supplementation or excessive use of calcium for eggshell formation [25], especially during peak and post-peak production as hen age progresses. Although the relationship between osteoporosis and keel bone damage is unclear, osteoporosis could cause a decline in the mineralized structure of bones and greater fragility and fracture, which would affect the keel bone [31]. Furthermore, genotype may be an important factor affecting keel bone damage via osteoporosis [32]. In this study, the Atak-S hens developed keel bone damage at an earlier stage and showed greater damage of the keel bone compared with the Atabey hens. Thus, the Atak-S genotype seems to have an increased tendency to develop keel bone damage. This could be attributed to higher egg weight with a larger egg surface area of brown Atak-S eggs [11], requiring more calcium for eggshell formation compared with white Atabey eggs. Similar findings have been reported by Graveland and Berends [33] and Whitehead [34]. The results in the present study suggested a correlation between keel bone damage and feather pecking in both genotypes. A study by Riber and Hinrichsen [35] also found a positive link between keel bone damage and plumage damage in laying hens housed in a free-range system.

This could be attributed to severe feather pecking being painful to the recipient bird, increasing fearfulness in the hens and resulting in fewer controlled movements due to escape attempts and flightiness [36]. Considering the possibility for increased escape behavior in victims of feather pecking, these individuals may have had an increased prevalence of keel bone fractures due to a higher possibility of falls or collisions in the house [34].

## 5. Conclusions

In conclusion, this present study found some significant differences in behavioral time budget and welfare status of the Turkish layer genotypes Atak-S (brown egg layer) and Atabey (white egg layer), and thus in suitability of these genotypes for free-range systems. In general, the Atak-S hens had more health problems than the Atabey hens. Furthermore, as the Atabey hens previously have been found to have increased egg production [11], this genotype seems to be more advantageous in free-range systems with outdoor access. However, the fearfulness of the Atabey hens should be considered, and further studies are needed to investigate whether the fearfulness of Atabey hens influences range use, particularly when kept in standard commercial flock sizes.

## Figures and Tables

**Table 1:** The ethogram used in the present study, including the definitions of the different behaviors modified by Zhao et al. [11].

Behaviors	Definition
Eating	Hen has its beak in contact with feed repeatedly/once
Drinking	Hen has its beak in contact with drinkers or raises its head when swallowing water
Preening	Hen has its beak in contact with its own plumage, performing movements of pecking, combing, rotating, or nibbling once or repeatedly
Feather pecking	Hen pecks the feathers of conspecifics
Walking–standing	Hen moves with a normal or quick speed or stands in a stationary position
Explorative pecking	Hen pecks other object in the house, except feathers
Resting	Hen lies on its abdomen or sits with its legs under the body

**Table 2:** Percentage of different behaviors observed for the Turkish laying hen genotypes Atak-S and Atabey in a free-range system.

Main Factors	Eating	Drinking	Preening	Feather Pecking	Walking–Standing	Explorative Pecking	Resting
Genotype							
Atak-S (brown)	18.4 ^a^	5.2 ^a^	2.8 ^b^	5.6 ^a^	19.1 ^b^	8.0 ^a^	41.0 ^b^
Atabey (white)	17.1 ^b^	3.7 ^b^	3.8 ^a^	5.1 ^b^	22.2 ^a^	5.3 ^b^	42.9 ^a^
SEM	1.2	1.4	0.5	0.3	0.4	0.2	0.4
Age (wk)							
24	16.6 ^c^	3.4 ^c^	3.1	4.4 ^b^	19.4 ^b^	6.0 ^b^	47.3 ^a^
40	17.5 ^bc^	4.6 ^b^	3.3	5.5 ^a^	20.5 ^ab^	6.7 ^ab^	42.0 ^b^
56	18.1 ^ab^	4.7 ^b^	3.3	5.8 ^a^	21.2 ^a^	6.7 ^ab^	40.4 ^c^
72	18.8 ^a^	5.2 ^a^	3.6	5.8 ^a^	21.4 ^a^	7.2 ^a^	38.2 ^d^
SEM	1.2	1.4	0.5	0.3	0.4	0.2	0.4
*p*-values							
Genotype	<0.001	<0.001	<0.001	0.001	<0.001	<0.001	<0.001
Age	<0.001	<0.001	0.143	<0.001	0.002	0.007	<0.001
Genotype × Age	0.092	0.183	0.823	0.915	0.957	0.122	0.093

*n* = 70 laying hens/pen. ^a–d^ Means in the column with different letters differ significantly (*p* < 0.05).

**Table 3:** Results of the tonic immobility (TI) tests for the Turkish laying hen genotypes Atak-S and Atabey in a free-range system.

Main Factors	TI Duration (Seconds)	Number of TI Inductions
Genotype		
Atak-S (brown)	103.2 ^b^	1.2 ^a^
Atabey (white)	120.4 ^a^	1.1 ^b^
SEM	2.8	0.01
Age (wk)		
24	107.5 ^ab^	1.1 ^c^
40	105.5 ^b^	1.1 ^bc^
56	117.4 ^a^	1.1 ^ab^
72	116.9 ^a^	1.2 ^a^
SEM	2.8	0.01
*p*-values		
Genotype	<0.001	<0.001
Age	0.012	<0.001
Genotype × Age	0.205	0.814

*n* = 12 laying hens/pen. ^a–c^ Means in the column with different letters^1^ significantly (*p* < 0.05).

**Table 4:** Clinical welfare indicators assessed at 24, 40, 56, and 72 weeks of age in the Turkish laying hen genotypes Atak-S (brown) and Atabey (white) in a free-range system.

Item	Weeks			
	24	40	56	72
Comb pecking wounds ^1^				
Atak-S	0.69	1.33	1.50	1.83
Atabey	0.31	0.67	0.83	1.33
SEM	0.2	0.2	0.3	0.2
*p*-values	<0.001	<0.001	<0.001	<0.001
Plumage damage (total) ^2^				
Atak-S	0	1.50	2.33	4.00
Atabey	0	1.33	0.50	1.33
SEM	-	0.2	0.5	0.6
*p*-values	-	0.250	<0.001	<0.001
Skin lesions ^1^				
Atak-S	0	0.37	0.67	1.17
Atabey	0	0.17	0.33	0.67
SEM	-	0.1	0.2	0.2
*p*-values	-	<0.01	0.021	<0.001
Footpad dermatitis ^1^				
Atak-S	0	0.17	0.53	1.50
Atabey	0.16	0.33	0.50	0.67
SEM	0.1	0.1	0.3	0.3
*p*-values	0.001	0.006	0.250	<0.001
Hock burns ^1^				
Atak-S	0.33	0.50	1.33	1.67
Atabey	0.17	0.50	0.50	1.17
SEM	0.1	0.2	0.3	0.2
*p*-values	0.0006	0.925	<0.001	<0.001
Toe damage ^3^				
Atak-S	0.17	0.50	0.67	1.03
Atabey	0.00	0.17	0.50	0.70
SEM	0.1	0.2	0.1	0.2
*p*-values	0.001	<0.001	0.022	<0.001
Keel bone damage ^3^				
Atak-S	0	0.33	0.67	1.00
Atabey	0	0	0	0.67
SEM	-	0.1	0.3	0.3
*p*-values	-	0.204	<0.001	0.001

*n* = 70 laying hens/pen. ^1^ Score 0, 1, to 2 denotes none to increasing evidence of lesions. ^2^ Total score of plumage condition is sum of breast, wing, and tail scores, each of which is 0, 1 or 2 (no wear, moderate wear, or featherless). ^3^ Score is 0 for absence and 1 for presence of damage.

## Data Availability

All data sets collected and analyzed during the current study are available from the corresponding author on fair request.
